# Mask use among pedestrians during the Covid-19 pandemic in Southwest Iran: an observational study on 10,440 people

**DOI:** 10.1186/s12889-020-10152-2

**Published:** 2021-01-14

**Authors:** Zahra Rahimi, Gholam Abbas Shirali, Marzieh Araban, Mohammad javad Mohammadi, Bahman Cheraghian

**Affiliations:** 1 Hearing Research Center, Department of Biostatistics and Epidemiology, School of Public Health, Ahvaz Jundishapur University of Medical Sciences, Ahvaz, Iran; 2 Department of Occupational Safety and Health Engineering, School of Public Health, Ahvaz Jundishapur University of Medical Sciences, Ahvaz, Iran; 3 Department of Health Education and Promotion, School of Public Health, Ahvaz Jundishapur University of Medical Sciences, Ahvaz, Iran; 4 Department of Environmental Health Engineering, School of Public Health AND Air Pollution and Respiratory Diseases Research Center, Ahvaz Jundishapur University of Medical Sciences, Ahvaz, Iran

**Keywords:** COVID-19, Face mask, Pedestrian, Iran, Observational study, Prevalence

## Abstract

**Background:**

Many countries have recommended the use of face masks for general population in public places to reduce the risk of COVID-19 transmission. This study aims to estimate the prevalence of face mask usage and investigate about different types of face mask and their distribution among pedestrians in southwest Iran during the Covid-19 pandemic.

**Methods:**

This cross-sectional study was conducted in August 2020 in Ahvaz, southwest Iran. Using a multistage sampling method, a total of 10,440 pedestrians selected from 8 urban districts and 92 neighborhoods of the city. The data gathered by observation method. Percentage, mean and standard deviation were used to describe the variables. Chi-square test, fisher exact test and Chi-square for trend used to assess relationship between two categorical variables. We used unconditional logistic regression model to control confounders.

**Results:**

The most common age group was 10 to 39 years and 67.9% of the participants were male. The overall prevalence of face mask usage was 45.6% (95% CI, 44.6–46.5). In general, as the age increased, the prevalence of face mask use significantly increased (*p* for trend < 0.001). Women used face masks significantly higher than men (60.2% vs. 38.7%, *p* < 0.001). Among the pedestrians who used the mask, 75.6% wore facemask correctly. The most common type of facemask used by the pedestrians were surgical (medical) masks (63.8%). In total, the prevalence of facemask usage was significantly higher during a.m. (49.4%) compared to p.m. (43.9%), (*p* < 0.001). Besides, in our study, 1.7 and 0.3% of Pedestrians had worn gloves and shielded respectively. Women used shields and gloves significantly higher than men (3.6% vs. 0.7%, *p* < 0.001). Also, women used shields more than men (0.5% vs. 0.3%, *p* = 0.036).

**Conclusion:**

We concluded that the prevalence rate of face mask use in Ahvaz was fairly low especially in men and younger people. Hence, the observed rates probably cannot protect the community against COVID-19 spread. Therefore, it is important to implement educational programs as well as to establish laws and regulations governing the use of face masks in public places.

## Background

Severe acute respiratory syndrome corona virus 2 (SARS-CoV-2) or corona virus disease 2019 (COVID-19) was initially reported from Wuhan, China on 31 December 2019. The World Health Organization (WHO) declared COVID-19 to be a pandemic on 11 March 2020. It is an ongoing global pandemic now [1]. According to the current evidence, Corona virus is mainly transmitted between individuals via respiratory droplets and contact routes, primarily from person to person during coughing, sneezing, talking [2].

Because there is not any effective treatment or vaccine against COVID-19 yet, personal protective measures including personal protective equipment (PPE) like masks, respirators (i.e. N95 or FFP2), shields and gloves can be used to prevent the infection [3, 4]. Social distancing and maintaining hand hygiene are the key strategies to prevent COVID-19 transmission (rational) whereas effectiveness of face mask usage by the healthy people in the community against COVID-19 is controversial, though increasingly recommended [5, 6]. The WHO had not yet recommended mass masking for healthy individuals in the all communities to prevent transmission of COVID-19 in its interim guidance of April 6, 2020, although it suggests the general public to wear a fabric mask in communities with widespread transmission, and especially in settings where physical distancing is difficult to maintain [2]. In the other hand, US Centre for Disease Control (CDC) recommends that people wearing masks in public places to reduce the risk of COVID-19 transmission, when around people outside of their household, especially in settings that social distancing cannot be maintained [4]. However, the use of mask alone is not sufficient to protect a person against COVID-19. It is also necessary to maintain a minimum physical distance from others, frequently washing hands and to avoid touching face (2). Although it is partly unknown the degree that masks protect against droplets/aerosols of respiratory system, [6] but even with a limited protective effect, face masks can reduce the risk of transmission of COVID-19 in the general public [3, 7–9] . They also can presumptively diminish the viral load, resulting to decrease the severity and risk of the death [10].

Masks have some protective effects including: protection of healthy person wearer in contact with an infected individual, source control (worn by a symptomatic or asymptomatic infected individual to prevent onward transmission) and remind others to continue practicing physical distancing [2, 8, 11]. The most common types of masks including surgical (medical) masks, Filtering Facepiece Respirators (FFR) or respirators like N95 or FFP2 and non-medical (Fabric) mask [2, 12]. The WHO recommended medical masks for using at health care facilities, people aged 60 or over, people of any age with underlying health conditions (including chronic respiratory disease, cardiovascular disease, cancer, obesity, immunocompromised patients and diabetes mellitus), anyone who is feeling unwell (including people with mild symptoms, such as muscle aches, slight cough, sore throat or fatigue), anyone awaiting COVID-19 test results or who has tested positive and people caring for someone who is a suspected or confirmed case of COVID-19 outside of health facilities. In the other hand, it suggests non-medical masks to use by general public under the age of 60 and who do not have underlying health conditions [13].

Masks in point of view filtration efficiency and breathability, can be different. Hence, it is important to use each type of mask in proper setting and situation. Medical masks and respirators are recommended to provide care to suspected or confirmed COVID-19 patients, not in public settings whereas wearing non-medical (Fabric) masks are recommended in public settings [2, 4].

Iran is among countries with the highest rates of morbidity and mortality due to COVID-19 [14]. A national official report announced incidence rate of COVID-19 in Ahvaz is among the highest in Iran [15]. The use of masks in tropical regions of Iran may be less welcomed due to problems caused by hot and humidity climate. This study aims to estimate the prevalence of face mask usage and investigate about different types of face mask and their distribution among pedestrians in Ahvaz, southwest Iran during the Covid-19 pandemic. This study also aims to assess the acceptance rate of the face mask practice worn by pedestrians. We believe these preliminary findings will help policymakers, managers and health professionals to design and implement their interventional programs.

By presenting the distribution pattern of mask use, findings of this study can lead to identification of high risk population groups and areas. We expect that the findings of this study to be used by health system policymakers to conduct health education interventions for the target groups, set preventive regulations for the community, and purposefully supply free masks.

## Methods

### Type of study

This population-based cross-sectional study was conducted during 10 days, from 2 to 11 August 2020 in Ahvaz, southwest Iran. A total of 10,440 pedestrians selected from 8 urban districts and 92 neighborhoods of the city. Pedestrians mask use behavior was accessed via observation. The data gathering was based on observation of passers-by in street because the observation method usually is more accurate and more valid than the self-reporting approach for assessing behaviors. This study was approved by Ethics Committee of Ahvaz Jundishapur University of Medical Sciences (IR.AJUMS.REC.1399.396).

### Inclusion and exclusion criteria

Inclusion criterion for this study was all pedestrians ≥2 years old in the city. Exclusion criteria for the study were: 1) fully covered face so that the observer cannot detect whether pedestrian wears the mask and, 2) be exposed to the observer for a short time so that it is not possible to record the required information.

### Study setting

**Fig. 1:**
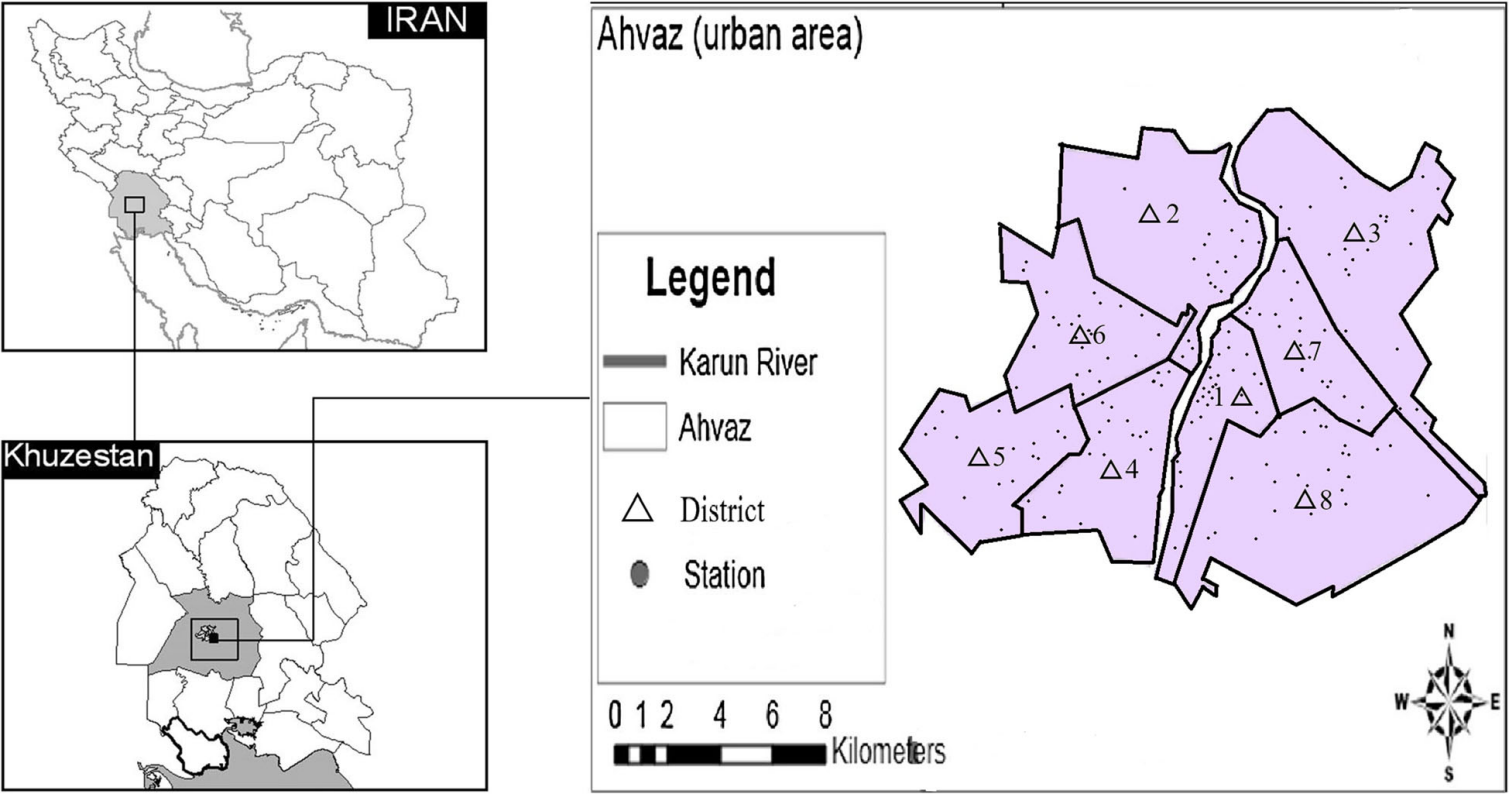
The map of Iran, Khuzestan province, Ahvaz city including the observation stations (The map is the authors’ own photo)

The *metropolitan city of* Ahvaz is located in the southwest Iran. Ahvaz is the capital of Khuzestan province. It’s population according to the 2019 census is 1,292,752. Ahvaz has a subtropical hot desert climate with long summers and short winters. The temperature sometimes exceeds 50 °C during summers and the humidity in sometimes reaches more than 90%. The map of Ahvaz including the observation stations showed in Fig. 1.

### Data collection

In this study, we employd eight observers with bachelor’s and master’s degrees in health and behavioral sciences. The main researcher of the project held a three-hour training session on the principles of observation, how to select the subjects and how to complete the checklist with the aim of standardizing the working method of the project. To ensure the quality of data collection, two Ph. D degree supervisors accompanied the observers for the first clusters. There was also continuous daily monitoring on observer performance by the supervisors. At the end of each day checklists collected by Supervisors were checked and observers were given feedback if there was any problem.

Observation stations were determined according to urban divisions of the city and allocated proportional to the population size, living in each district and neighborhood. These stations were determined from detailed maps of urban divisions and were selected from the crowded passages of each neighborhood. At each station, data of 60 pedestrians were collected including gender, approximate age, use of facemask, gloves and shield, type of facemask, and correct use of facemask. Insufficient coverage of the mouth and nose, wearing facemask upside down or inside-out were defined as “incorrect or unacceptable” use of the mask. Observation was performed during the busy hours of each area from 9.00 to 13.00 and 17.00 to 23.00.

### Sample size and sampling method

In order to determine the minimum sample size, we used the formula for estimating a population proportion. For this purpose, α = 0.05, *p* = 0.5, d = 0.04 and a design effect equal to 1.6 were considered. A minimum sample size of 960 estimated for each district. Regarding to the unequal size of the districts and using a proportional to size sampling method, the final sample size needed for this study was estimated 10,440 pedestrians. In total, 174 clusters of 60 people from 93 urban neighborhood of Ahvaz were assessed in this study.

We used a Multistage sampling method in this research. At the first stage, each of 8 urban districts was considered as a stratum. Then defined number of clusters were assigned to each neighborhood. Each cluster was consisting of 60 pedestrians. The location of the observation stations was determined by a targeted sampling strategy from the busy passages of each neighborhood. A non-probability convenience sampling method was used for the last stage. In such a way that at each observation station, after deploying the observer, the closest pedestrian to the observer was selected as the first sample and entered to the study. After finished recording the data of the first person, the next closest person to the observer selected as the next sample and this was continued until the total number of selected persons in each cluster reach to 60.

### Statistical analysis

Descriptive statistical measures including mean, standard deviation and percent used to describe the data. The estimated prevalence rates presented with 95% confidence interval (95% CI). Chi-square test, fisher exact test and Chi-square for trend used to assess relationship between two categorical variables. We used unconditional logistic regression model to control potential confounders. Odds ratios used to assess strength of the associations. The Statistical significance was declared if the *p*-value was less than 0.05. The analyses were carried out with SPSS version 22.

### Ethics

The Ethics Committee of Ahvaz Jundishapur University of Medical Sciences (AJUMS.REC.1399.396) confirmed the morality and ethics of the study.

## Results

A total number of 10,440 pedestrians were assessed in terms of facemask usage. The most common age group was 35 to 39 years old. Among the studied individuals, 7072(67.9%) were male. Demographic characteristic and frequency of personal protective measures among of the assessed pedestrians are shown in Table 1. Among the observed pedestrians, 4749 people had used facemasks. The overall prevalence of facemask usage was 45.6% (95% CI, 44.6–46.5). The prevalence rates of facemask usage by the assessed factors and their 95% confidence intervals are presented in Table 2. The highest prevalence of facemask usage was seen in the age group of 60 years and older, and the lowest was seen in the age group of less than 10 years, 61.7 and 26.6% respectively.

**Table 1:** Demographic characteristic and frequency of personal protective measures among pedestrians in Ahvaz

Variable		n	(%)
**Age group**	0–9 y	719	6.9
	10–39 y	6678	64.2
	40–59 y	2592	24.9
	60 y and older	412	4.0
**Sex**	Male	7072	67.9
	Female	3336	32.1
**Area**	East	5760	55.2
	West	4680	44.8
**Urban district**	One	1680	16.1
	Two	1140	10.9
	Three	1380	13.2
	Four	1140	10.9
	Five	960	9.2
	Six	1440	13.8
	Seven	1200	11.5
	Eight	1500	14.4
**Face mask use**	Yes	4749	45.6
	No	5673	54.4
**Type of face mask**	Surgical mask	3030	63.8
	Cloth mask	940	19.9
	Filtered mask	731	15.4
	Other	43	0.9
**How to use a facemask**	correct use	3586	75.6
	Uncovered mouth and/or nose	598	12.6
	inside-out	470	9.9
	upside-down	90	1.9
**Gloves use**	Yes	176	1.7
	No	10,246	98.3
**Shield use**	Yes	35	0.3
	No	10,405	99.7
**Time**	a.m.	3120	29.9
	p.m.	7320	70.1

**Table 2:** Prevalence rates of face mask usage by sex, age group, urban district and area

	Number of observed pedestrians	Face mask usage	
		n	(CI 95%) prevalence
**Overall prevalence**	10,422	4749	45.6 (44.6 to 46.5)
**Sex**			
Male	7063	2734	38.7 (37.6 to 39.9)
Female	3336	2009	60.2 (58.5 to 61.9)
**Age group**			
0–9 y	719	191	26.6 (23.4 to 30.0)
10–39 y	6678	3015	45.1 (43.9 to 46.4)
40–59 y	2592	1276	49.2 (47.3 to 51.2)
60 and older	412	254	61.7 (58.6 to 66.4)
**Urban district**			
One	1673	809	48.4 (45.9 to 50.8)
Two	1140	725	63.6 (60.7 to 66.4)
Three	1377	684	49.7 (47.0 to 52.3)
Four	1138	606	53.3 (50.3 to 56.2)
Five	960	307	32.0 (29.0 to 35.0)
Six	1439	301	20.9 (18.1 to 22.2)
Seven	1197	430	35.9 (33.2 to38.7)
Eight	1498	887	59.2 (56.7 to 61.7)
**Area**			
East	5745	2810	48.9 (47.6 to 50.2)
West	4677	1939	41.5 (40.0 to 42.9)

**Fig. 2:**
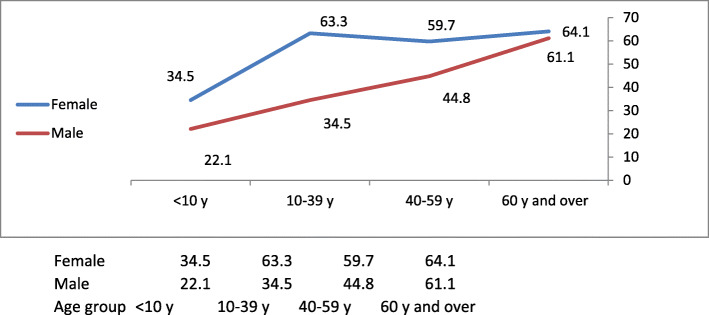
Comparison of age trends of mask usage by gender

In general, as the age increased, the prevalence of facemask usage significantly increased too (*p* for trend < 0.001). This trend was obviously seen for men but women showed different pattern so that facemask usage in age group under 10 was low while the prevalence of facemask usage among the other age groups were higher and almost the same (Fig. 2). In total, women used facemasks significantly higher than men (60.2% vs. 38.7%, *p* < 0.001).

The pedestrians in the eastern area of Ahvaz city worn facemasks significantly more than the western area (48.9% vs. 41.5%, *p* < 0.001). The prevalence of facemask usage among pedestrians of the eight districts of Ahvaz were highly different, so that the highest prevalence was observed in district two and the lowest was seen in district six, 63.6 and 20.9% respectively. The prevalence of facemask usage was even more different among the neighborhoods. It ranged between 1.7 to 78.3%.

The most common type of facemask used by the observed pedestrians were surgical (medical) masks (63.8%) and the lowest were the filtered masks including N95 respirators (15.4%). Older pedestrians wore filtered masks more than younger pedestrians while younger pedestrians used cloth mask more frequently (*p* = 0.002). However, we did not find any significant association between the type of used facemasks and gender (*p* = 0.44).

Among the pedestrians who used the mask, 75.6% wore facemask correctly, 12.6% did not cover completely their mouths and noses, 9.9% wore inside-out and 1.9% wore upside-down. Acceptable facemask practice was significantly higher in women than men (78% vs.73.9%; *p* = 0.017) whereas there was no statistically significant difference between the age and correct wearing of facemasks (*p* = 0.19). Besides, in our study, 1.7 and 0.3% of Pedestrians had worn gloves and shielded respectively. Women used gloves significantly higher than men (3.6% vs. 0.7%, *p* < 0.001) also wearing gloves was higher in older people (*p* = 0.033). Also, women used shields more than men (0.5% vs. 0.3%, *p* = 0.036) but no significant association was found between use of shields and age (*p* = 0.34) (Table 3).

**Table 3:** Association between personal protective measures and sex among pedestrians in Ahvaz

Variables	Male n (%)	Female n (%)	Total n (%)	***p***-value*
**Type of face mask**				
**Surgical mask**	1741 (63.7)	1289 (64.2)	3030 (63.8)	0.44
**Cloth mask**	540 (19.7)	400 (19.9)	940 (19.8)	
**Filtered mask**	446 (16.3)	285 (14.2)	731 (15.4)	
**Other**	8 (0.3)	35 (1.7)	43 (0.9)	
**Total**	2735 (100)	2009 (100)	4744 (100)	
**How to use facemask**				
**Correct use**	2020 (73.9)	1566 (78.0)	3586 (75.6)	0.017
**Uncovered mouth and/or nose**	391 (14.3)	207 (10.3)	598 (12.6)	
**Inside-out**	267 (9.7)	203 (10.1)	470 (9.9)	
**Upside-down**	58 (2.1)	32 (1.7)	90 (1.9)	
**Total**	2735 (100)	2009 (100)	4744 (100)	
**Gloves use**				
**Yes**	48 (0.7)	128 (3.6)	176 (1.7)	< 0.001
**No**	6778 (99.3)	3460 (96.4)	10,238 (98.3)	
**Total**	6826 (100)	3588 (100)	10,414 (100)	
**Shield use**				
**Yes**	18 (0.3)	17 (0.5)	35 (0.3)	0.036
**No**	7054 (99.7)	3319 (99.5)	10,373 (99.7)	
**Total**	7072 (100)	3336 (100)	10,408 (100)	

*The *P* values used in this table were obtained by chi-square test

**Fig. 3:**
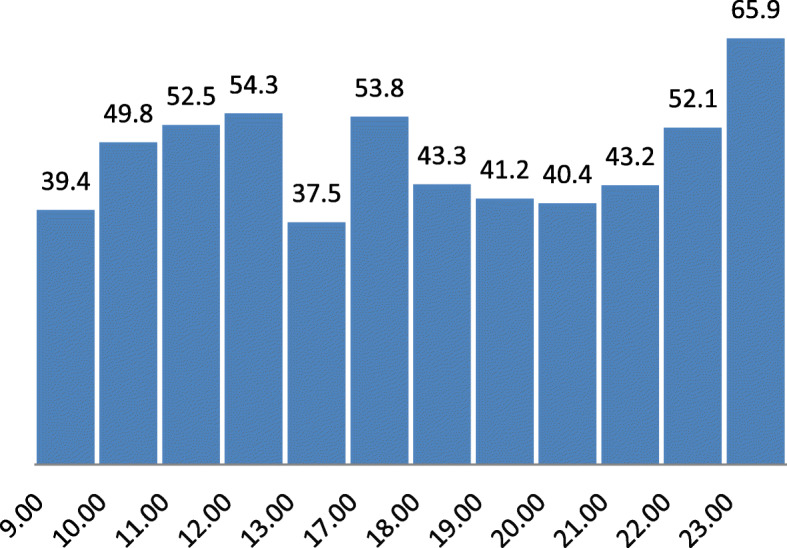
The prevalence rates of facemask usage at the different hours

The observations and data gathering were occurred between 9:00 o’clock to 23.00 o’clock. The lowest prevalence of facemask usage was seen at 13.00 (37.5%) while the highest was observed at 23.00 (67.9%). In total, the prevalence of facemask usage was significantly higher during a.m. (49.4%) compared to p.m. (43.9%), (*p* < 0.001). The prevalence rates of facemask usage at different times of day are presented in Fig. 3.

Because age and sex could play confounder role in this assessment, unconditional logistic regression model was performed to control effects of these potential confounders. After controlling for age and sex, we observed a significant association between prevalence of facemask usage and time of observations so that the odds of facemask usage during a.m. was 26% higher than p.m. (Odds Ratio: OR = 1.26; 95% CI, 1.16–1.38; *p* < 0.001). The results are presented in Table 4.

**Table 4:** The crude and adjusted odds ratios for effects of time on face mask prevalence using multiple logistic regression model

Variables	Crude ORs	***P***-value	Adjusted ORs	***P***-value
**Age group**				
0–9 y	0.23 (0.17–0.29)	< 0.001	0.19 (0.14–0.24)	< 0.001
10–39 y	0.37 (0.31–0.45)		0.35 (0.29–0.42)	
40–59 y	0.44 (0.37–0.52)		0.41 (0.35–0.49)	
60 y and older	1		1	
**Sex**				
Male	1	< 0.001	1	< 0.001
Female	0.42 (0.38–0.45)		0.39 (0.36–0.43)	
**Time**				
a.m.	1	< 0.001	1	< 0.001
p.m.	1.24 (1.14–1.35)		1.26 (1.16–1.38)	

## Discussion

At present, COVID-19 has sparked a pandemic and is spreading rapidly in many countries [16]. Because there is no vaccine or effective treatment for this disease, conducting interventions such as use of facemask, social distancing and washing hands are urgently needed to limit the transmission. Despite the WHO primary recommendations against universal masking, emphasis on this strategy is increasing in the world. In Iran, the law on the use of masks in public places, government offices and banks was implemented on June 4.

In this study the prevalence of facemask usage was low (45.6%). This rate has been inefficient to control the disease hence, Ahvaz was in a critical situation and in the red zone for several months.

A survey conducted from February 25 to April 25 found that 64% of the public reported wearing a mask and gloves in crowded places [17]. In the present study the prevalence of facemask usage was much lower than the rates from Hong Kong study among pedestrians [18], Malaysia study among hospital visitors [19] and Malaysia study on general public at wet markets 94.8, 96.9 and 99.7% [20], Singapore 90%, In India 81–84%, In the United Arab Emirates between 78 and 81%, In Saudi Arabia 72% [17], Pakistanis (85.8%) [21] respectively. Similar to our finding observed in Bangkok airport (46%) [22]. The prevalence rates of face mask use in India, Kerala [23], Lima, Paris, Boston and Atlanta airports (41, 27, 4, 3 and 2% respectively) [22] were much lower than Ahvaz. The observed differences can be due to demographic and cultural characteristics of the assessed population, different methods of data gathering, policy of the governments about mass masking and the risk of COVID19 transmission in the countries.

In our study the highest prevalence of face mask usage was in the age group of 70 years and older (71.7%). Our result showed the prevalence of face mask usage increased with age. Similar findings were reported among the elderly in Japan (aged 60–69) [24] and Australia (aged 65–74) [25], the percentages face mask usage were 43.6 and > 60%, respectively. This may be due to the perception of higher risk of morbidity and death due to COVID19 for higher age groups.

Besides, the prevalence of face mask use in women was significantly higher than men (60.2% vs. 38.7%, *p* < 0.001). This could be due to the fact that women generally pay more attention to their health status and making healthy behaviors. Conversely, the prevalence rates observed in Malaysia studies showed no difference in both sex [19, 20].

The observed differences in prevalence rates of face mask usage were impressive among the districts and neighborhoods of Ahvaz. This could be mostly due to the differences in socio-economic status. The low socioeconomic level usually leads to low health literacy and public awareness, lack of access to masks, as well as low purchasing power.

The most common type of mask in our study was surgical mask (63.8%). The same finding reported by Gunasegaram et al. [19, 20] and Tam et al. [18]. This can be questionable because the WHO and CDC did not recommend the use of surgical masks in general population [2, 26]. In contrast, they recommended using cloth masks in public setting. This type of masks can be easily manufactured or made at home and reused after washing [27] and it is more affordable than other masks. Besides, we found that the types of face masks were differently used among the age groups. Filtered masks were used higher by older pedestrians while younger people used cloth face masks much higher than the older pedestrians. People in higher age groups usually fill more risk of COVID-19 so they may be use more frequently filtered masks with the purpose of their higher protection. A Survey of the Healthcare Workers in Afghanistan showed that Participants had access to one-layer medical masks produced inside Afghanistan 378 (41%), medical masks 451 (49%), N95 masks 206 (22%) [28].

Our findings showed higher prevalence rates of face mask usage during am hours in relation to pm hours, that it may be due to weather conditions especially the higher temperatures in the afternoon. The similar finding reported by Cheng et al. [5].

Wearing properly a mask is necessary to get the maximum protection against COVID19 [29]. In this study, acceptable rate of using masks among the observed pedestrians was 75.6%. The percentage of acceptable face mask practice in our study was lower than some similar studies. This rates were reported from Malaysia about 95.63 and 88.75% [19, 20] and from Hong Kong about 87% [18]. Besides, we found that the correct practice of face mask use in women was higher than men. This can be due to better following the health protocols by women.

Our study had a number of limitations. Due to use of observation method for the data gathering, we could not assess some important factors like socioeconomic status and the reasons for not wearing masks. Besides, we did not ask exact age of the subjects and approximate ages were recorded instead. Therefore, a non-differential misclassification can be occurred in the age grouping.

This investigation had some major strengths. Using observation method for data gathering in this study can leads to more valid data in comparison to use of questionnaires and self-reporting method. Furthermore, our large sample size guaranteed sufficient statistical power and precise estimation of the rates so that the calculated confidence intervals are mostly narrow.

### Direction for future research

Future studies assessing motivations for or against mask wearing will be valuable, particularly if collected across different countries and national cultures.

## Conclusion

In summary, we found that the overall prevalence rate of face mask usage in Ahvaz was fairly low especially in men. Hence, the observed rates of mask usage probably cannot protect the community against COVID-19 spread. Therefore, it is important to plan and conduct educational programs to promote healthy behaviors in the community, especially for the high risk groups. Besides, to establish laws and regulations governing the use of face masks in public places is necessary for increasing the rate of mask population coverage.

## Data Availability

Upon request, we can offer onsite access to external researchers to the data analyzed at Ahvaz Jundishapur University of Medical Sciences, Ahvaz, Iran. To do so, Dr. Cheraghian should be contacted.

## Abbreviation

COVID-19: Corona virus disease 2019
